# Copy number variation and brain structure: lessons learned from chromosome 16p11.2

**DOI:** 10.1186/s13073-015-0140-8

**Published:** 2015-02-16

**Authors:** Jason L Stein

**Affiliations:** Neurogenetics Program, Department of Neurology, Center for Autism Research and Treatment, Semel Institute, David Geffen School of Medicine, University of California, Los Angeles, Los Angeles, CA 90095 USA

## Abstract

Recent work has linked specific genetic variation found in human populations to risk for developing neuropsychiatric diseases. How that risk is mediated through molecular-, cellular- and systems-level mechanisms now becomes the central question in this field. Two recent papers studying high-penetrance copy number variation at chromosome 16p11.2 find large changes in brain structure, refining hypotheses about the regions of the brain that are affected and implicating specific neurodevelopmental processes in these changes.

## The long road

Research in the field of genetics has identified many types of genetic variation - from single base-pair changes to large chromosomal deletions and duplications - that have a statistical association with increased risk for a disease. These are a huge boon, especially to neuropsychiatric research, suggesting a causal basis for many of these disorders for the first time. But identification of a risk variant is just the first step; genetic variants exert their effects at molecular, cellular, circuit and systems levels to alter the function of the brain, which can then manifest as an illness. Variant functionality is largely unexplored and is the next great frontier in human genetics research. The hope is that by disentangling a variant’s biological consequences, we may be able to interfere with its downstream effects to develop therapeutic treatments that adjust dysfunctional biochemical pathways. However, it is a long road from identifying genetic risk variants to a mechanistic understanding of a neuropsychiatric disease.

One of the mutational classes that contribute to neurodevelopmental disorders are copy number variations (CNVs), defined as >1 kilobase regions that harbor deletions or duplications of chromosomes. What is striking is that at certain spots in the genome, CNVs are found significantly more frequently in patients with neuropsychiatric disease than in controls. One particular locus, at chromosome 16p11.2, has been strongly associated with a variety of neuropsychiatric phenotypes, and the attempt to elucidate the biological consequences of variations at this locus is now beginning. Two recent studies have analyzed the effects of 16p11.2 variants on brain anatomy and have shed light on the processes that may lead to disease [1,2].

## The 16p11.2 variant

Copy number changes at 16p11.2 are rare in people with neuropsychiatric diseases and even rarer in healthy populations (duplication and deletion rates in healthy populations are approximately 0.04% [3,4]). When these mutations do occur, they increase risk for a wide variety of disorders, including autism spectrum disorder (ASD), schizophrenia, developmental delay, epilepsy and obesity [3-7]. The mutations are not fully penetrant; that is, not everyone with a mutation will also have the disease [4]. However, people with 16p11.2 deletions have an approximately nine times greater likelihood of developing ASD, but no appreciable increase in risk for schizophrenia; those with the duplication have a nine times greater likelihood of developing both ASD and schizophrenia [3]. Variation at this genomic region thus represents a clear risk factor for a range of neuropsychiatric disorders and provides insight into their molecular basis. The functional effects of such variations are not limited to neuropsychiatric phenotypes: 16p11.2 deletion carriers are much more likely to be overweight, whereas duplication carriers are more likely to be underweight [6].

The large genomic region either deleted or duplicated in these CNVs at 16p11.2 spans 29 genes. Molecularly, gene expression within the CNV has been shown to follow the cardinality of the mutation [6,8]; that is, people with duplications have increased expression and deletion carriers have decreased expression of genes within the region. Interestingly, the expression of genes outside the region is also affected and those genes are often involved in synaptic function, chromatin modification, or are other known ASD risk genes [8]. This implies a shared mechanism at the molecular level across different etiologies of ASD.

## The anatomy of a CNV

Given the neuropsychiatric risk and previously identified associations with head circumference [6], a clear next step is to determine if and how brain structure, as measured through magnetic resonance imaging (MRI), is affected in patients carrying 16p11.2 CNVs. Because this is a rare variant with high penetrance, it is useful for the study of known broad-spectrum disease phenotypes in relatively small sample sizes and in largely control populations; in turn, this has the advantage of enabling researchers to investigate the effect of the variation without their results being confounded by differences in medication or the altered environment of a patient. Two studies [1,2] have recently measured macro-scale brain structure via MRI in individuals with 16p11.2 deletion (*N* = 27, *N* = 14, respectively) and duplication (*N* = 17, *N* = 17, respectively), most of whom had not been diagnosed with either schizophrenia or ASD. Given the rarity of the mutation and the hypothesized large effects on the brain, these should be considered relatively large sample sizes.

Both studies find large global differences in intracranial volume and total white and gray matter volumes; deletion carriers have larger volumes and duplication carriers have smaller volumes relative to controls. Both studies find an effect in the same direction on cortical surface area, but less evidence is found for alterations in cortical thickness. This dichotomy is consistent with a developmental change in the formation of the brain. The radial unit hypothesis predicts that this type of abnormality in cortical surface area could be due to a greater number of neural progenitors being produced during fetal development in deletion carriers, which then differentiate to create a cortical plate with a larger surface area [9]. The replicable findings of high effect across two cohorts give strong support to this developmental mechanism. In addition, these studies identify a phenotype that stem cell or animal models of the 16p11.2 mutation can attempt to replicate, and subsequently to correct through drug screening.

When studying chromosomal effects on the structure of specific brain regions, the picture becomes more complicated. One of the strongest findings was an effect on thalamic volume, which was greater in deletion carriers and smaller in duplication carriers in both studies, even after controlling for a global measure of head size (intracranial volume). Overall cerebellar volume showed the same relationship in one study [1], but specific regions of the cerebellum were found to have the opposite direction of effect in the other [2]. The volume of regions of the striatum had the same relationship with carrier status as thalamic volume in one study [2], but this was not significantly replicated in the other study [1]. Regional, rather than global, thickness and area in specific cortical areas were not evaluated in one study [1], so it is currently not possible to assess the reproducibility of this phenotype. It should be noted that differences in the method of analysis, age of participants and genetic variation outside the 16p11.2 region, and within the region in an unaffected chromosome, could be responsible for the different results seen across the cohorts in the two studies. Obtaining a clearer picture of the specific regions affected in patients will take larger cohorts.

## Further down the road

These two studies are an excellent example of collaborative consortium-based science at work. Because 16p11.2 mutations are so rare, organizations such as the Simons Variations in Individuals Project and the 16p11.2 European Consortium aggregate individuals from many sites around the world to acquire enough subjects to obtain the statistical power to perform analyses like these. This collaborative framework allows the discovery of novel insights into rare genetic influences on brain structure and how they lead to disease. Similar efforts like the Enhancing Neuroimaging Genetics through Meta-analysis (ENIGMA) consortium [10] are revealing how common variants affect brain structure, and may also lead to increases in mechanistic understanding about the links from genes to brains to disease, and everything in between. It is also critical that, given how resource intensive imaging studies on genetically defined cohorts are, they should be performed in such a way that the data be easily shared and analyzed by other investigators with minimal burden. This has not been the standard of practice in neuroimaging, as it is in genomics and genetics, but the differing findings in the two studies discussed here emphasize how useful this would be. Ultimately, such studies may help to delineate how genetic variation leads to neuropsychiatric diseases through alterations in brain structure.

## Abbreviations

ASD: Autism spectrum disorders
CNV: Copy number variation
ENIGMA: Enhancing neuroimaging genetics through meta-analysis
